# Frequency of poor quality of life and predictors of health related quality of life in cirrhosis at a tertiary care hospital Pakistan

**DOI:** 10.1186/1756-0500-5-446

**Published:** 2012-08-20

**Authors:** Om Parkash, Romaina Iqbal, Fatima Jafri, Iqbal Azam, Wasim Jafri

**Affiliations:** 1 Department of Medicine, Aga Khan University Karachi, Karachi, Pakistan; 2 Community Health Sciences, Aga Khan University Karachi, Karachi, Pakistan; 3 Medical College, Aga Khan University, Karachi, Pakistan

**Keywords:** Hepatitis, Chronic liver disease, Hepatic encephalopathy, Ascites, Quality of life, Upper GI bleed, Cirrhosis, Health related Quality of life, Hepatitis C and Hepatitis B

## Abstract

**Background:**

Cirrhosis produces variety of symptoms which eventually lead to a negative impact on Health Related Quality of Life (HRQOL). The general aim of this study was to evaluate the magnitude of poor HRQOL and to assess factors related with HRQOL in patients with CLD in Pakistan.

**Findings:**

This was a cross sectional study conducted in gastroenterology outpatient clinics of Aga Khan University Hospital, Karachi on adult patients with cirrhosis. In this study chronic liver disease questionnaire (CLDQ) was used to assess HRQOL of these patients and CLDQ score was used as an outcome measure to determine factors related with HRQOL.

273 participants were recruited in the study; 155 (57%) were males. Mean age of participants was 49 ± 11 years. The most common cause for cirrhosis was viral infection 247(91.5%). Mean Model for End Stage Liver Disease (MELD) score was 12.6 ± 6.8 and 2/3 of patients 209 (76.6%) had advanced cirrhosis in Child Turcot Pugh (CTP) B or C stage. Poor HRQOL was seen in 187(69%; 95% C.I.: 63%, 74%) of the participants. Mean CLDQ score was 4.36 ±1.1. Amongst all of the domains, fatigue domain had lower CLDQ score. Hemoglobin (β = 0.09 [SE = 0.04]), Albumin (β = 0.32[SE = 0.09]), Diastolic Blood Pressure (DBP) (β = 0.01[0.005) prior history of decompensation (β = 0.98[SE = 0.39] were significant factors associated with HRQOL in patients with liver cirrhosis.

**Conclusion:**

Frequency of poor health related quality of life determined by CLDQ score is high in patients with liver cirrhosis. Hemoglobin, serum albumen, prior history of decompensation (like encephalopathy and upper gastro intestinal bleed), are associated with health related quality of life.

## Findings

### Background

Recently Quality of Life (QOL) has become the principal goal of medical care because of the increasing emphasis on the patients as focal point of health care, patients’ functioning preservation and wellbeing. Henceforth measurement of patient’s HRQOL is receiving attention in medical research [1].

HRQOL is a subjective multidimensional concept which includes functional status, emotional and social wellbeing as well as general health [2]. Similarly World Health Organization (WHO) has also expanded and redefined the definition of health which now includes mental and social wellbeing, therefore WHO has defined health as ‘state of complete physical, mental and social well-being and not merely the absence of disease or infirmity’ [3,4]. This relatively new concept of HRQOL as part of psychosocial outcome has been integrated as a common approach along with traditional biomedical outcome in the last two decades of the previous century and since then it has become an important outcome measure in the assessment of disease management [5]. There has been a rapid development in the concept of HRQOL due to growing awareness of the importance of understanding the effect of health related intervention on patients’ routine life, rather than only on treatment of their bodies. Because this concept is important for those who live with less optimism of cure such as decompensated liver cirrhosis patients therefore HRQOL is important in terms of physical, and psychosocial wellbeing for patients; suffering from chronic debilitating illnesses [6].

The measurement of HRQOL is especially essential in patients with cirrhosis where recovery is not always achievable and HRQOL is reduced. In Pakistan this is the first attempt (to the best of the investigators’ knowledge) to assess HRQOL and elucidate factors associated with it in CLD patients.

The general aim of this study was to assess HRQOL in patients with liver cirrhosis. We also aimed to assess factors (such as age, gender, education, job, etiology of CLD, history of decompensation of cirrhosis like ascites or PSE or GI bleed; CTP score/Class, MELD score and socioeconomic status) related with HRQOL in liver cirrhosis patients. The Chronic Liver Disease Questionnaire (CLDQ) is a specific HRQOL tool that has been developed by Younossi *et al.* in 1999 on 133 patients [7]. CLDQ is a validated chronic liver disease specific instrument and can be used for all types of liver diseases to get significant information about CLD related HRQOL [8].

## Materials and methods

This was a cross sectional study conducted at gastrointestinal (GI) outpatient clinic of Aga Khan University Hospital Karachi from May 2009 to March 2010. Aga Khan University Hospital is a private, tertiary care hospital in Karachi Pakistan. This University Hospital has 563 beds in operation and provides services to over 50,000 hospitalized patients and to over 600,000 outpatients annually with the help of professional staff and facilities that are among the best in the region. Ethical permission was obtained from the Aga Khan university ethics review committee.

All patients who had established diagnosis of liver cirrhosis of any etiology and fulfilling inclusion criteria were recruited in study after obtaining the informed consent. The inclusion criteria include all adults age ≥14 years, either history of viral CLD (HBsAg or HCV) or non viral CLD, history of any of clinical features of cirrhosis like ascites, encephalopathy or upper GI bleed, any of the laboratory features including prolonged prothrombin time, decreased albumen and total bilirubin and any of ultrasonographic evidence of CLD including shrunken liver, dilated portal vein, splenomegaly. We used nonrandom purposive technique for recruiting participants in the study. We did not include individuals with concomitant comorbids like heart failure, chronic renal failure (CRF), chronic obstructive pulmonary disease (COPD), any malignancy like cancer of the lungs etc., and inflammatory bowel disease (Ulcerative colitis and Crohn’s disease). Once the patients consented, their interviews were conducted in the gastroenterology clinic room. The baseline information of demographics, education, socioeconomic status, marital status, etiology of CLD, severity of CLD in terms of child score was recorded from the medical record file of study subjects at the time of interview in clinic visit. The routine clinical examination, laboratory parameters such as complete blood count, prothrombin time (PT) with international normalized ratio (INR), liver function tests (LFT), Albumen, serum creatinine (Cr), blood urea nitrogen, electrolytes (Na, K, Cl, Hco3) and ultrasound features of cirrhosis were recorded from participants’ hospital file on the predesigned proforma. HRQOL was assessed by using CLDQ questionnaire and all the questions in CLDQ were verbally asked in native language if they could not understand English.

### CLDQ questionnaire

This is a disease specific type of HRQOL which consists of 29 questions and are assessed on a seven point Likert scale and responses range from ‘All of the time to none of the time’. Higher score on the questionnaire is indicative of minimum symptoms and lower score indicates poorer symptoms. This instrument incorporates the emotional, social, physical and mental health and is divided into 6 domains; Abdomen symptoms (AS), Fatigue (FA), Systemic symptoms (SS), Activity (AC), Emotional function (EF) and Worry (WO). Each domain has different number of questions like abdomen and activity domain has 3 questions each, fatigue, worry and systemic domain has 5 questions, and worry domain has 6 questions. These questions are randomly distributed. We followed the scoring system devised by Younossi *et al.* (2001).

Data was analyzed on SPSS-16 version. We made categories of CLDQ score for determination of frequency of HRQOL and these categories were good if mean CLDQ score ≥5 and bad if CLDQ score is <5 [7]. Categories were made for CLDQ score by assigning the values for each category based on the international available literature that had shown mean CLDQ score of >5 in normal participants [3,7]. We also used CLDQ score as continuous variable for regression analysis. Categories were made for age, occupation, education, socioeconomic class [9,10].

Frequencies and percentages (e.g. Child class or etiology of CLD) were used for categorical variables, and mean and standard deviation for continuous variables (e.g. CLDQ score, MELD score). Student *t* test was used to assess difference in mean CLDQ score between males and females, while one way analysis of variance (ANOVA) was used to compare mean difference of CLDQ score for different categories; education classes, occupation, prior history of decompensation (like Upper Gastrointestinal bleed, Hepatic Encephalopathy and Ascites) of cirrhosis/CLD, income classes, etiology of CLD and Child Classes (p value <0.05 was taken as significant). Simple linear regression was also used to determine the relation between CLDQ score and the independent variables.

Variables found to be significant in simple linear regression with a p value of <0.25 were included in multiple linear regression analysis. These variables were prioritized based on their F change and R^2^ change. Final model was selected based on high F change with significant P value

## Results

Total of 273 participants were recruited in the study; among them 155 (57%) were males; mean age of participants was 49 years (SD ± 11 years); among them majority of study participants i-e 184 (67.5%) belonged to age group of 40–60 years. Other demographic attributes are shown in Table 1. In this study most common cause for cirrhosis was viral infection 247 (91.5%). Mean CTP score was 8 ± 1.85 and mean MELD score was 12.6 ± 6.8. Two thirds of participants 209 (76.6%) had advanced cirrhosis (CTP B or C). Poor HRQOL was seen in 187 (69%) of the participants. Mean score of the CLDQ was 4.36 (SD ± 1.1). Amongst all of the domains, fatigue domain had significantly lower CLDQ score. Hb (β = 0.09 [SE = 0.04]), Albumin (β = 0.32 [SE = 0.09]), diastolic blood pressure (DBP) (β = 0.01[0.005), and prior history of decompensation (β = 0.98[SE = 0.39] were found to be significant factors associated with liver cirrhosis. Education was also forced into the final model as it showed significant confounding (change in β = 32%).

**Table 1:** Demographic characteristics of participants with cirrhosis

**Variables**	**Number of participants (%)**
Mean age of participants ( ±SD)	49(SD ± 11 years)
Age group*	
<40 years	55(20.1%)
41-50 years	98(35.9%)
51-60 years	86(31.5%)
>60 years	34(12.5%)
**Gender**	
Male	155(57%)
Female	118(43%)
**Occupationˆ**	
Labor	66(24%)
Employee	72(26%)
House Wife	105(39%)
Retired	30(11%)
**Socioeconomic!**	
Low income	95(35%)
Middle income	148(54%)
High Income	30(11%)
**Education Status**	
Uneducated	45(17%)
Primary (5 years)	63(23%)
Secondary(10 years)	80(29%)
Higher secondary & above (>10 years)	85(31%)

*Sumskiene J *et al.*, World J Gastroenterol. 2006 Dec 28;12(48):7792–7.

ˆMitraZandi *et al.* Health and Quality of Life Outcomes 2005;3:35.

!Federal Bureau of Revenue website and T. Jaffar *et al.* 2009 Annal of Internal Medicine.

### Clinical parameters

Two hundred forty seven (90.5%) had viral related cirrhosis. Overall HCV related cirrhosis is the most common cause of cirrhosis in 198 (72.5%) participants while HBV related cirrhosis was only in 49 [Isolated HBV related in 39 (14.3%) participants and combined HBV/HDV related in 10 (3.7%) participants] and only 26 (9.5%) had non B non C related cirrhosis. Mean child score was 8 ± 1.85 with 140 (51.3%) had child class B, 69 (25.3%) had child class C and 64 (23.4%) had child class C. Mean MELD score was 12.6 (6.8).

Only 93 (34%) participants had history of any decompensation [Ascites 40 (14%), UGI bleed 45 (16.5%) and hepatic encephalopathy 8(3%)] and majority of participants about 180 (66%) had no history of cirrhosis related decompensation.

Study participants’ haemodynamics were essentially normal with pulse of 77.64 ± 11 b/min, systolic blood pressure of 112.64 ± 16 mmHg, respiratory rate of 17.05 ± 4.76 br/min and temperature of 36.99 ± 0.60 ^0^C

### Chronic liver disease questionnaire score

Details CLDQ score is given in Table 2, in which it is clearly seen that among all domains fatigue domain had significantly lower mean CLDQ score of 4.36 (SD ± 1.1). Majority of the patients (n = 187, 69%; 95% C.I.: 63%, 74%) had poor (<5) CLDQ score.

**Table 2:** CLDQ Score

**Variable**	**Minimum**	**Maximum**	**Mean (SD)**	**Algorithm for assigning score**
CLDQ domains*				Sum of all questions in each domain
Abdomen	3	21	13.47 (5.14)	
Fatigue	5	35	19.26 (6.3)	
Systemic	5	35	20.98 (6.47)	
Activity	3	21	13.14(5.25)	
Emotion	8	56	35.76(11.08)	
Worry	6	35	23.92(7.68)	
Domain Mean CLDQ score				Score obtained by dividing total domain score with number of questions
Abdomen	1	7	4.48(1.71)	
Fatigue	1	7	3.85(1.27)	
Systemic	1	7	4.19(1.29)	
Activity	1	7	4.38(1.75)	
Emotion	1	7	4.47 (1.38)	
Worry	1	7	4.78 (1.53)	
Mean CLDQ	1.3	6.98	4.36 (1.15)	Score obtained by dividing number of domains or Total score/29
Total CLDQ	38.00	202	126.54 (32.97)	Sum of all questions

*Domains: (N = indicates the number of questions in CLDQ questionnaire).

Abdominal (AB) = Sum of N1 + N5 + N17.

Fatigue (FA) = Sum of N2 + N4 + N8 + N11+ N13.

Systemic (SY) = Sum of N3 + N6 + N21 + N23 + N27.

Activity (AC) = Sum of N7 + N9 + N14.

Emotion (EM) = Sum of N10 + N12 + N15 + N16 + N19 + N20 + N24 + N26.

Worry (WO) = Sum of N18 + N22 + N25 + N28 + N29.

ABM = AB/3.

FAM = FA/5.

SYM = SY/5.

ACM = AC/3.

EMM = EM/8.

WOM = WO/5.

CLDQM = SUM (OF ABM FAM SYM ACM EMM WOM)/6.

On multivariable linear regression, Hb (β = 0.09 [SE = 0.04]), Albumin (β = 0.32 [SE = 0.09]), diastolic blood pressure (DBP) (β = 0.01[0.005), and prior history of decompensation (β = 0.98[SE = 0.39] were found to be significant factors associated with CLDQ score in patients with liver cirrhosis (Table 3). Interactions were checked for biologically plausible variables child class, MELD, income class, education and sodium. No significant interaction was found. Same variables were then checked for confounding. Out of these education was found to have significant confounding (β change =32%). Hence education was then forced into the final model.

**Table 3:** Factors determining CLDQ score on multivariable regression*

**Variables**	***β***	**Std. Error**	**95% CIˆ**
(Constant)	1.465	.550	0.382 ; 2.548
Albumen	.302	.099	0.107; 0.497
Diastolic B.P	.013	.005	.003; 0.023
Haemoglobin	.090	.041	.009; 0.171
Primary Education^1^	.347	.199	−0.045; 0.739
Secondary Education	.244	.201	−0.152; 0.64
Higher Secondary Education and above	-.121	.209	−0.544; 0.291
Upper GI bleed^2^	.322	.181	−0.035; 0.678
Hepatic encephalopathy	.982	.390	0.214; 1.75
Ascites	.158	.192	−0.22; 0.536

*Coefficient of determination (R2) =0.156.

ˆ CI = Confidence Interval.

1 = Uneducation (reference category).

2 = No history of decompensation (reference category).

### Laboratory parameters

Among the Laboratory parameters are these patients had low mean Hb of 10.65 ± 1.65 gm/dl and low platelets with median value 87000 (IQR 87; 135). This cohort of patients also had low protein and with low albumen. Details are shown in Table 4.

**Table 4:** Laboratory Parameters for patients with cirrhosis attending a tertiary care hospital

**Variables**	**Mean (SD)**
**Haematological Parameters**	
Haemoglobin (gm/dl)	10.65 (1.65)
White cell count(per cmm)	6.08 (3.28)
Platelets *	*87.00 (87;135)
Prothrombin Time(Sec)	15.80 (4.79)
**Biochemistry**	
Total Protein (gm/dl)	6.75 (1.32)
Albumen (gm/dl)	2.61 (0.71)
Creatinine (mg/dl)	1.10 (0.57)
Sodium (Meq/L)	134 (5)
Potassium (Meq/L)	3.97 (0.62)
Chloride (Meq/L)	106 (9.6)
Bicarbonate (Meq/L)	20.46 (3.74)
Liver function tests*	**Median (IQR)**
Total Bilirubin (mg/dl)	1.5 (1;2.95)
SGPT (IU/L)	39 (25;58)
SGOT (IU/L)	59 (40;92)
GGT (IU/L)	48 (30;81)
ALK Phosphate (IU/L)	110 (79;166)

*Median and Inter Quartile Range.

## Discussion

This is the first study conducted at a tertiary care hospital in Pakistan, that suggests that majority of the patients with CLD had poor QOL as assessed by CLDQ score. This study also found that albumen, diastolic blood pressure; haemoglobin and prior history of decompensation are significantly related with HRQOL in patients with CLD.

Globally Cirrhosis/Chronic liver disease (CLD) caused by many entities, is responsible for major mortality and morbidity [7]. Liver cirrhosis is one of the commonest conditions for which individuals are hospitalized in Pakistan where viral related cirrhosis is the most common cause [11-13]. Therefore it is important to understand the HRQOL in such a debilitating condition especially in Pakistan where liver transplant facility is not available. Literature regarding HRQOL is scarce in the world and especially from Pakistan.

Among the viral causes; HBV associated cirrhosis had lower CLDQ score as compared to HCV or HBV/HDV associated cirrhosis. However overall the number of chronic hepatitis B patients as compared to patients with hepatitis C was small. This is different from a study [14], in which they had shown less impairment of quality of life in patients suffering from HBV associated cirrhosis as compared to other causes of cirrhosis like HCV or Cholestatic associated cirrhosis. In an Italian study that used different questionnaires like SF-36 and Nottingham Health Profile Questionnaire (NHP) for assessing HRQOL compare to our study but it also demonstrated that HRQOL was reduced in patients with liver cirrhosis [15]. Another study by Foster *et al.*, which has reported that CLDQ score is more affected in patients with Chronic Hepatitis C (CHC) associated cirrhosis hence concluded that the symptoms caused by CHC are qualitatively different from those associated with CHB cirrhosis [16]. Hence more studies are required to confirm the poor quality of life in patients with Hepatitis B.

Results in this study have shown that mean CLDQ score is not significantly different among different age groups. Hence this suggests that increasing age does not affect the mean CLDQ score as also shown by Sumskiene in their study [3]. Younossi *et al.* had suggested that older age affects the QOL in patients with cirrhosis [5]. However, this result cannot be compared with Younossi *et al.* because they had enrolled all types of CLD patients including patients with hepatocellular carcinoma (HCC) whereas this study had enrolled only liver cirrhosis participants.

Our findings about gender are similar to those reported by Sumskiene *et al.*[3]. On comparison of the mean CLDQ score in different categories of CTP class lower CLDQ score was seen in child class C as compared to Child class A. This result is comparable with the study by Sumskiene *et al.* (Lithuanian group) [3]. Sumskiene group had also seen that HRQOL in cirrhosis worsens with increasing of severity of liver cirrhosis that is from better HRQOL (Higher CLDQ score) in CTP A to poor HRQOL (Lower CLDQ score) in CTP C. Younossi *et al.* had also suggested that as disease severity worsens in chronic liver disease, HRQOL is affected [5]. In this study, results have shown that neither MELD score nor CTP score was an independent predictor of CLDQ score. Similarly in a study conducted in North America which revealed that HRQOL was not affected by the severity of disease and these results are comparable with our findings too in terms of severity of life affecting the quality of life [17]. We also observed that people who belong to poorer income class had lower CLDQ score. This finding is similar to a study conducted in Thailand in which it was observed that people with low income had poor quality of life [18]. It is plausible that low income may be associated with low education status and delayed detection of CLD because of late help seeking behavior of less literate participants, it is an assumption which needs further studies actually. The low income might have also caused poor nutritional status that can also affect the QOL.

The mean global CLDQ score is comparable with scores reported by other international studies for liver cirrhosis [7,19]. A Thai study had shown that mean CLDQ score decreases as disease severity worsens from compensated group (5.2 ± 1) to decompensate group (4.5 ± 1.2). The Thai study had divided these patients with chronic hepatitis and CTP A into compensated, while patients with CTP B and C into decompensated CLD. The mean CLDQ score which was reported by Thai group is also comparable with this study’s mean CLDQ score though slightly lower as compared to decompensated CLD. However, mean CLDQ score of compensated CLD was slightly higher in Thai study because they had also recruited chronic hepatitis patients while in this study only cirrhotic patients were recruited [18]. However Younossi *et al.* had reported lower mean CLDQ score as compared to this study that can be explained because Younossi had also included patients with more advanced disease including patients with hepatocellular carcinoma (HCC) [5]. This variability among different studies is because of heterogeneous group of patients being studied in different studies. Other reason for difference in CLDQ score might be because of different socioeconomic status and socio-demographic attributes of study participants.

In this study an independent association of CLDQ score with various factors of cirrhosis like Alb, Hb, DBP, prior history of decompensation especially history of upper GI bleed along with history of HE and education was seen. Hence this study has identified certain factors like Hb and Alb which can be corrected and ultimately will help in better HRQOL and moreover these parameters can also be used in the design of randomized control trials to assess their utility in HRQOL. Different studies have been conducted to determine factors associated with liver cirrhosis but none had shown any consistent factors for HRQOL. A recent study by Les at al (n = 212) cirrhotic patients in Barcelona Spain had done study to identify the potential treatable factors associated with liver cirrhosis. Spanish study had determined that factors like prior history of decompensation, non-alcohol etiology (viral and others), hypoalbumenia, decrease in hemoglobin and female gender are independent predictors of HRQOL [19]. Low hemoglobin (Anemia) may lead to symptoms like tiredness, shortness of breath, mild pedal edema, low grade fever, which depend upon severity of anemia. Low hemoglobin as a determinant of HRQOL had also been seen in other diseases like chronic renal failure and congestive heart failure [20,21]. Our study has shown that low Hb had affected the HRQOL. Low HB in liver cirrhosis is multifactorial; hypersplenism, poor nutritional intake because of diet restriction imposed on such patients by community as well by physicians and history of upper as well as lower GI bleed in them. With this study we can assume that HRQOL can be improved with rise in HB either by transfusion wherever necessary or by giving therapies such as either erythropoietin or iron supplements like in other diseases [22]. Low albumen usually indicates poor nutritional status and associated with low CLDQ score by impairing the certain domains of CLDQ (Fatigue, activity, physical functioning and vitality) by usually causing Ascites [19].

We observed that prior history of decompensation especially upper GI bleed was associated with CLDQ score. Any history of decompensation suggestive of fairly advanced stage of CLD therefore HRQOL among such patients would be low. Hence it can be emphasized that timely management of such complications would lead to higher CLDQ score that ultimately would translate into better HRQOL and improve the survival as well [23]. An interesting finding our work is that diastolic blood pressure is also an independent predictor of mean CLDQ score. Till to date association between diastolic blood pressure and CLDQ score has not been reported. However, over the last two decades researchers have been trying to see the hemodynamic effects and arterial compliance in patients with liver cirrhosis. As this is established fact that liver cirrhosis does cause major hemodynamic changes in body which include abnormal distribution of blood flow and volume, that lead to cardiovascular dysfunction, alteration in rennin angiotensin system and effect on systemic vascular resistance [24,25]. Studies have assessed overall arterial compliance in patients with liver cirrhosis but none of these have highlighted the importance of maintaining normal diastolic blood pressure [24,25]. Another study done on cirrhotic patients to determine the 24 hour blood pressure recording because these patients have abnormal systemic haemodynamics with reduced arterial blood pressure. In their study they had determined that Cirrhotic patients have raised pulse, but interestingly these patients had normal arterial pressure during night time. They had also determined that circadian rhythm in blood pressure and pulse was diminished. Their results may suggest a major defect in the ability of optimal regulation of blood pressure in cirrhotic patients [26]. Despite this we are not sure whether this is just a statistical association or there is some biological plausibility for diastolic blood pressure. This issue needs further research.

### Limitations

Since this is a cross sectional study, we cannot make conclusions about causality (eg low Hb causes low CLDQ score etc.) we can only suggest that these factors are associated with CLDQ score. Also majority of the participants had chronic hepatitis C (CHC) so these results cannot be corroborated to other causes of CLD hence causing limited external validity. This study has limited external and internal validity because a very selective group of patients with well compensated cirrhosis and only outpatients from one tertiary care hospital were enrolled but based on our findings a larger study, involving several hospitals can be conducted to confirm our findings. Another limitation was that patients with advanced hepatic encephalopathy were not enrolled because of their inability to give consent and answer the questions. In fact hepatic encephalopathy is a major determinant of HRQOL in patients with CLD.

## Conclusion

We conclude that majority of the patients with CLD had poor QOL as assessed by CLDQ score. We have also determined that hemoglobin, serum albumen, prior history of decompensation like encephalopathy and upper gastrointestinal bleed are associated with HRQOL of life as suggested by CLDQ score therefore HRQOL can be improved if these factors are taken care of on time.

## Recommendations

It is recommended that study evaluating HRQOL in patients with cirrhosis and healthy controls should be done to determine the true prevalence of QOL impairment associated with liver cirrhosis in a large population based study. A further improvement on this recommendation would be to develop a longitudinal follow-up study on such patients.

## Abbreviations

QOL: Quality of Life; HRQOL: Health Related Quality of Life; CLD: Chronic liver disease; CLDQ: Chronic Liver Disease Questionnaire; CTP: Child Turcot Pugh; MELD: Model for End Stage Liver Disease; WHO: World Health Organization; GI: Gastrointestinal; CRF: Chronic renal failure; COPD: Chronic obstructive pulmonary disease; PT: Prothrombin Time; LFT: Liver function tests; INR: International Normalized Ratio; Cr: Creatinine; AS: Abdomen symptoms; FA: Fatigue; SS: Systemic symptoms; AC: Activity; EF: Emotional function; WO: Worry; HBV: Hepatitis B virus; HCV: Hepatitis C virus; HDV: Hepatitis Delta virus; DBP: Diastolic blood Pressure; HCC:Hepatocellular Carcinoma; CHC: Chronic Hepatitis C.

## Competing interests

The author declared that they have no competing interest.

## Authors’ contribution

OP has made the initial proposal in consultation with RI and WJ. IA has helped us in providing the assistance in statistical aspect including the sample size. RI and WJ had reviewed the proposal and give approval to submit to ethics committee. WJ has provided the clinical supervision and helped in final drafting the article. IA has given support in statistical analysis and helps in sample size calculations. FJ helped in data collection. Final draft with corrections was made by OP and RI. In revised version OP, RI, IA and WJ has reviewed the comments and helped OP in final drafting especially IA has helped in responding to statistical observations. All authors read and approved the final manuscript.
